# Tunisian Pediatricians’ Attitudes and Practices Toward COVID-19 Immunization and Other Vaccines

**DOI:** 10.3390/ijerph22020233

**Published:** 2025-02-06

**Authors:** Ines Cherif, Rabeb Gharbi, Ghassen Kharroubi, Walid Affes, Jihene Bettaieb

**Affiliations:** 1 Laboratory of Medical Epidemiology, Institut Pasteur de Tunis, University of Tunis El Manar, Tunis 1002, Tunisia; ines.cherif@pasteur.tn (I.C.); rabebgharbi25@gmail.com (R.G.); ghassen.kharroubi@pasteur.tn (G.K.); walidaffes1996@gmail.com (W.A.); 2 Mediterranean and Black Sea Programme in Intervention Epidemiology Training (MediPIET), European Centre for Disease Prevention and Control (ECDC), 171 83 Stockholm, Sweden; 3 Laboratory of Transmission, Control and Immunobiology of Infections (LR11IPT02), Institut Pasteur de Tunis, University of Tunis El Manar, Tunis 1002, Tunisia

**Keywords:** vaccines, pediatricians, attitude, practice, COVID-19, Tunisia

## Abstract

Pediatricians are among the most trusted sources of vaccine information for parents. We aimed, in this study, to describe the attitudes and practices of Tunisian pediatricians regarding non-National Immunization Schedule (NIS) vaccines, specifically the COVID-19 vaccination for children, and to identify factors associated with their willingness to recommend it. We conducted a national cross-sectional study among Tunisian pediatricians between July and October 2023 using a standardized questionnaire administered face-to-face. We calculated prevalence with 95% confidence intervals (95%CIs) and adjusted odds ratios (aOR) using multivariable logistic regression. Of 330 contacted pediatricians, 192 (58.2%) responded (mean age: 50.9 ± 12.9 years). The majority (89.1%, 95% CI: [84.6–93.5]) said that they recommend other vaccines that are not part of the NIS and 40.6% [33.7–47.6] declared their willingness to recommend the COVID-19 vaccination for children. The odds of pediatricians willing to recommend the COVID-19 vaccination for children were higher among those who believed that this vaccine would reduce school absenteeism (aOR = 2.3 [1.1–5.1]) and among those who have great confidence in the Ministry of Health’s recommendations regarding COVID-19 vaccination (aOR = 6.1 [2.2–16.9]). More than half of the pediatricians in Tunisia recommend other vaccines that are not part of the NIS but show hesitancy toward the COVID-19 vaccine. Thus, involving pediatricians in the decision-making process for childhood vaccination strategies is crucial.

## 1. Introduction

Vaccination is considered one of the greatest achievements in public health. Over the past 50 years, global immunization programs have saved the lives of approximately 154 million individuals, reducing infant deaths by 40% globally and by 50% in the African region [1]. Even though the majority of countries around the world tend to recommend the same kinds of vaccines for children, there are still certain differences in immunization schedules from one country to another [2]. This is explained by differences in healthcare systems, structures, and resources, as well as variations in disease epidemiology [3].

As of March 2023, the Tunisian National Immunization Schedule (NIS) included tuberculosis, hepatitis B, hepatitis A, pneumococcal, a combination of rubella–measles, inactivated polio vaccine (IPV) and live attenuated oral polio vaccine, acellular pertussis, diphtheria, tetanus, and hemophilus influenza type b vaccines [4]. Adherence to childhood routine vaccines is high in Tunisia [5]. Nevertheless, there are other vaccines that are authorized in the country but are not part of the NIS. These vaccines are not covered financially by the government and are, thus, fully paid for by the parents.

One of the vaccines that does not figure in the NIS but was authorized to use in the country is the COVID-19 vaccine. Indeed, The World Health Organization (WHO) has authorized COVID-19 vaccination of children, initially for ages 12 to 15, then extending eligibility to ages 6 to 11 years, and finally to children from the age of 6 months in August 2022, while prioritizing those with co-morbidities [6]. Following this authorization, several countries initiated vaccination for children against COVID-19 with different age limits [7].

Tunisia authorized vaccination of adolescents aged between 12 and 14 in November 2021 and of children aged between 5 and 11 in September 2022 [8,9]. Nevertheless, by the end of October 2022, less than 10% of Tunisians under 15 years old had received one dose or more of the COVID-19 vaccine [10].

The decision by parents to vaccinate their children is complex and influenced by various factors, such as concerns about the vaccine safety, confidence in the healthcare system, and vaccine hesitancy among healthcare workers [11]. Indeed, pediatricians play an important role in influencing caregivers’ adherence to pediatric vaccination [12,13,14], as they are considered one of the most trusted sources of information on vaccines by parents [15]. It is, therefore, important to identify their attitudes regarding vaccines that are not included in the NIS, with a specific focus on the COVID-19 vaccine. In fact, understanding barriers to COVID-19 vaccine recommendation by caregivers is important for shaping vaccine awareness campaigns for future emerging diseases. Also, even though the epidemic situation of COVID-19 has changed and the number of severe cases has dramatically decreased, we are not safe from a new wave and the emergence of a new variant. Various studies have been conducted worldwide to assess attitudes regarding COVID-19 vaccination among adults [16]. For the pediatric population, some surveys have been conducted mainly among parents to assess their intention to vaccinate their children against COVID-19, revealing a high level of hesitancy among participants. Various factors influencing parents’ attitudes toward pediatric COVID-19 vaccination have been identified, including trust in vaccine safety and the healthcare system, concerns about the short duration of clinical trials, and the advice of doctors [17,18,19,20,21]. Few studies have been conducted among pediatricians, most of which demonstrate a high willingness among participants to recommend the COVID-19 vaccine for children [17,22,23,24]. However, to our knowledge, no studies of this kind have been conducted in Tunisia or North Africa. One of the primary factors influencing behavioral intentions, according to the Theory of Reasoned Action (TRA), is individuals’ attitudes toward the behavior, which include their perceptions of the benefits and risks associated with a vaccine [25]. Research employing this framework involves evaluating pediatricians’ attitudes toward vaccinations, including any hesitancy or concerns they may have. We aimed, in this study, to describe the attitudes and practices of Tunisian pediatricians, from July to October 2023, regarding vaccines that are not included in the NIS in general and the COVID-19 vaccination for children in particular, as well as to determine the factors associated with the pediatricians’ willingness to recommend the COVID-19 vaccination for children.

## 2. Materials and Methods

### 2.1. Study Design and Population

We conducted a national descriptive cross-sectional study among pediatricians practicing in both private and public (university hospitals, regional hospitals, primary healthcare facilities) sectors in Tunisia. This study took place over a period of four months, from July to October 2023. We included pediatricians who had a specialization certificate in pediatrics and who gave their consent to participate in it.

### 2.2. Sampling Design and Sample Size

Participants were selected based on a random sampling from the National Chamber of Physicians list published online.

The sample size was calculated with the OpenEpi Software Version 3.01 using the following settings:

- Target population size: 790 (according to the national chamber of physicians list)
- Precision: 0.05
- Prevalence of COVID-19 vaccine recommendation by pediatricians: 30% (based on a pilot study)

The minimal required sample size was 230. This sample size was then adjusted to a non-response rate of 30% giving a total of 329.

### 2.3. Data Collection

Data were collected face-to-face. Neither names nor surnames were collected, and a unique identification number was assigned to each participant.

The data collection tool was developed based on an in-depth literature review [12,13,15,22,23] and was reviewed and validated by the research team. A pilot study was conducted to train investigators (three physicians from the study team) and to assess the comprehensibility and clarity of the questions. Pediatricians were initially contacted by phone to inform them about the survey, seek their approval, and schedule an appointment. Those who agreed to participate were approached by trained investigators in their workplace to respond to the questions after giving their written informed consent.

The data collection tool included 26 items divided into three sections: demographic and professional characteristics of study subjects, pediatricians’ history of COVID-19 vaccination, and pediatricians’ attitudes and practices toward vaccines that are not included in the NIS in general and the COVID-19 vaccination for children in particular in Tunisia. Open-ended questions were used to determine the reasons for accepting or refusing to recommend the COVID-19 vaccine for children. General statements related to attitudes included 4-point Likert-type items (1: disagree, 2: neither agree nor disagree 3: agree, 4: I do not know) about COVID-19 vaccination efficacy and safety. Four-point Likert-type items were used to assess the degree of confidence of participants in the recommendations of the WHO and the Tunisian Ministry of Health (TMH) on COVID-19 vaccination (1: Not at all confident, 2: Not very confident, 3: Moderately confident, 4: Very confident).

### 2.4. Statistical Analysis

The paper questionnaires that were completed by the investigators were validated, entered, and analyzed using the Epi Info version 7.2.5.0 software (developed by the Centers for Disease Control and Prevention (CDC)). Simple frequencies and percentages were calculated for the qualitative variables and are presented with a 95% confidence interval (CI). Quantitative variables were summarized by means and standard deviations. Answers to the general assessment of the surveyed pediatricians’ confidence in the TMH and the WHO, initially using a four-degree Likert scale, were grouped as follows: “Not at all confident” and “Not very confident” in “No/Minimal confidence”, and other responses including “Moderately confident” and “Very Confident” in “Medium/High confidence”. Also, to make data interpretation easier in univariate and multivariable analysis, responses to the general COVID-19 statements, initially using a four-degree Likert scale, were grouped as follows: “Agree” and “Others”, which included “Neither agree nor disagree”, “I don’t know”, and “Disagree”.

To determine the factors associated with pediatricians’ willingness to recommend the COVID-19 vaccination for children, we first conducted a univariate analysis using the Pearson Chi 2(ﬡ2) test, or the exact Fisher test if the validity conditions were not met. Factors independently associated with pediatricians’ willingness to recommend the COVID-19 vaccine to children were identified using a binary logistic regression model. Explanatory variables with a degree of significance of less than 20% in univariate analysis were included in the initial multivariable model. The final model variables were selected manually using a stepwise backward procedure. We verified collinearity between the independent variables in the multivariable analysis model by examining the correlation matrix.

## 3. Results

A total of 330 pediatricians were contacted: 112 refused to participate in the study and 26 were unreachable. Thus, 192 answered the questionnaire, giving a response rate of 58.2%. The majority of non-respondents were female (60.1%), 84.1% of whom worked in the private sector and half of them worked in northern Tunisia. The response rates by region were as follows: 64.9% in the north, 56.3% in the center, and 32.6% in the south.

The mean age of the study population was equal to 50.9 ± 12.9 years, with extremes ranging from 30 to 82 years. Most participants were female (57.3%). Nearly three quarters (71.3%) of the surveyed pediatricians worked in the private sector, more than half (66.7%) had their workplace in northern Tunisia, 48.4% had a professional experience of 20 years and over, and 70.3% had children under 16 years old (Table 1). The majority of participants (64.6%) did not have any history of chronic illnesses.

### 3.1. Pediatricians’ Recommendation of Vaccines That Are Not Included in the NIS

**Figure 1:**
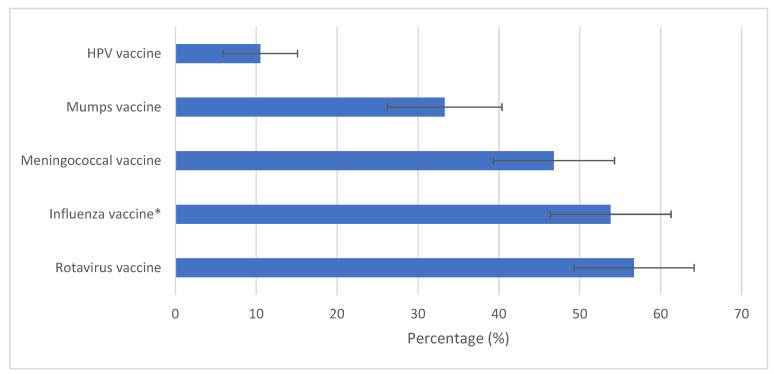
Vaccines recommended by the surveyed pediatricians outside of the national immunization schedule in Tunisia, July–October 2023 (N = 171). *: Influenza vaccine for children with chronic diseases. HPV: Human Papillomavirus.

The majority of participants (89.1%, 95% CI: [84.6–93.5]) replied that they recommend other vaccines that are not part of the NIS, mainly the rotavirus vaccine (56.7%) and the influenza vaccine for children with chronic illness (53.8%) (Figure 1).

Half of the participants (50.5% [43.4–57.6]) declared that they conduct vaccination-promoting actions in their workplace. Among them, 53.8% [43.7–63.7] utilize posters, while 38.5% [29.1–48.7] educate their patients about vaccine importance during medical visits.

### 3.2. Attitudes and Practices of Pediatricians Regarding COVID-19 Vaccination for Children

Less than half of the surveyed pediatricians (40.6% [33.7–47.6]) were willing to recommend the COVID-19 vaccine for children aged 16 years and below, 65.4% [54.3–75.0] of whom would recommend it only for those with chronic diseases. Among participants, 13.8% [8.9–18.8] stated that they see no reason that would prevent them from recommending the vaccine to children. For the remaining respondents, the most common reasons that may lead to the non-recommendation of the COVID-19 vaccination were fear of the vaccines’ side effects (38.2% [30.8–45.8]) and the absence of severe cases of COVID-19 among children (36.4% [29.0–43.8]). Regarding the willingness to recommend the COVID-19 vaccine for children, 21.3% [15.4–27.8] of the surveyed pediatricians stated that no reason would incite them to recommend the vaccine. For the others, the main reasons included protecting children from severe forms of the disease (40.5% [32.6–48.5]) and a potential worsening of the epidemic situation (28.4% [21.1–35.6]).

Among participants, 70.4% [63.9–77.0] did not support making the COVID-19 vaccine mandatory for children. Also, almost half of our participants agreed with the statement that the COVID-19 vaccine may lead to serious side effects among children (45.8% [38.8–52.9]) and that natural immunity following infection lasts longer than that provided by vaccination (44.0% [36.9–51.0]), as detailed in Table 2.

Overall, 31.1% [24.5–37.7] of the surveyed pediatricians reported having little to no confidence in the WHO recommendations on COVID-19 vaccination and 25.9% [19.7–32.2] in the TMH recommendations.

### 3.3. Factors Associated with Pediatricians’ COVID-19 Vaccine Recommendations to Children

Pediatricians working in the public sector were more willing to recommend the COVID-19 vaccine to children compared to those in the private sector (OR = 2.5 [1.3–4.7]). Additionally, pediatricians without children under 16 years old were more likely to recommend vaccination than those who have children in that age group (OR = 2.2 [1.2–4.2]), and those aged 40 and below showed a higher willingness to recommend the vaccine compared to their counterparts aged 60 years and over (OR = 2.5 [1.1–5.6]). However, gender was not significantly associated to pediatricians’ willingness to recommend the COVID-19 vaccine (*p* = 0.425).

Regarding the attitudes, pediatricians who had medium or high confidence levels in the TMH (OR = 9.1 [3.4–24.2]) and the WHO COVID-19 recommendations (OR = 8.3 [3.5–19.5]) were more willing to recommend the COVID-19 vaccine for children. Also, those who agreed with the statements that COVID-19 vaccine protects from severe diseases in children, prevents transmission of the virus to family members, reduces school absenteeism, and protects from long COVID were more likely to recommend the COVID-19 vaccine to children. Those who agreed with the statement that the COVID-19 vaccine can cause serious side effects in children were significantly less likely to be willing to recommend it (Table 3).

In multivariable analysis, the variables that were included in the initial model were the following: sector of practice, professional experience, having children under 16 years old, vaccinating children against COVID-19 protects them from SARS-CoV-2 infection, vaccinating children against COVID-19 is important to reduce school absenteeism, vaccinating children against COVID-19 protects them from long COVID, it is important to vaccinate children against COVID-19 to prevent its transmission to their families, and pediatricians’ confidence level in the TMH recommendations on COVID-19 vaccination.

Factors independently associated with pediatricians’ willingness to recommend the COVID-19 vaccine were confidence in the TMH recommendations on COVID-19 vaccination and agreement with the statement that the COVID-19 vaccine for children reduces school absenteeism, as detailed in Table 4.

## 4. Discussion

The majority of participating pediatricians said that they recommend other vaccines that are not part of the NIS, mainly the rotavirus vaccine (56.7%) and the influenza vaccine for children with chronic illnesses (53.8%). Regarding the COVID-19 vaccination for children, less than half of the pediatricians (40.6%) were willing to recommend it, among whom 65.4% would recommend it only for children with chronic diseases. In the multivariable analysis, factors independently associated with pediatricians’ willingness to recommend the COVID-19 vaccine included confidence in the TMH recommendation on COVID-19 and agreement with the statement that the COVID-19 vaccine for children reduces school absenteeism.

Outside of the NIS, the rotavirus vaccine was the most frequently recommended vaccine by pediatricians in our study. Likewise, a study conducted by Çatakli et al. [26] about the attitudes of physicians in Turkey concerning vaccines that are not included in the NIS found that the rotavirus vaccine was the most commonly recommended one and nearly half wanted it to be added in the Turkish NIS. The recommendation of the rotavirus vaccine by our participants is probably explained by the health burden of rotavirus gastroenteritis in Tunisia. Indeed, a study conducted in central-east Tunisia during the period 2009–2011 found that nearly a quarter of hospitalizations for acute diarrhea among children under 5 years old were due to rotavirus. The economic burden of hospitalizations attributed to the rotavirus infection was also estimated to be high [27]. The rotavirus vaccine is included in the national immunization program of several countries worldwide [28]. In order to advocate its introduction in the NIS among policymakers in Tunisia, it is important to enhance rotavirus data collection and to conduct vaccine-effectiveness and cost-effectiveness studies [29]. The second most recommended vaccine outside of the NIS was the influenza vaccine for children with chronic diseases. Indeed, the WHO Strategic Advisory Group of Experts (SAGE), as well as the Centers for Disease Control and Prevention (CDC), recommend a seasonal influenza vaccination for children aged 6 to 59 months, as they are considered at higher risk of severe influenza, especially those under two years and those with chronic conditions [30,31].

Regarding the COVID-19 vaccine, less than half of pediatricians were willing to recommend this vaccine to children. This percentage was lower than that shown in other studies that found that over half of healthcare providers recommend the vaccine to children [17,22,23]. Such a difference may be explained by the fact that these studies were conducted in 2021 and 2022, when the epidemic situation was more critical. The COVID-19 epidemic situation was characterized by an important number of hospitalizations and deaths, mainly by the circulation of the Delta variant in 2021 and by the beginning of the circulation of the Omicron variant in 2022. Indeed, the attitudes and practices of pediatricians may shift in response to fluctuating infection rates and the emergence of variants of concern (VOCs) with varying levels of pathogenicity [22]. Also, even though the WHO has authorized vaccination against COVID-19 for children and adolescents, they are considered as low priority-use groups. Nevertheless, those with chronic conditions were classified in the medium priority-use group due to the increasing risk of severe COVID-19 disease [32]. Indeed, more than half of pediatricians who were willing to recommend the COVID-19 vaccine stated that they would do so only for children with chronic health conditions.

Agreement with the statement that the COVID-19 vaccine for children reduces school absenteeism was independently associated with pediatricians’ willingness to recommend the COVID-19 vaccine for children. Indeed, there is evidence that children significantly contribute to the transmission of SARS-CoV-2, especially in school and household environments [33]. Additionally, minimizing school disruptions was one of the rationales stated by the WHO behind the authorization of vaccination for children [34]. Nevertheless, in the context of Omicron, the effect of the currently available vaccinations on lowering symptomatic disease and transmission was estimated as modest in the latest version of the WHO roadmap on uses of COVID-19 vaccines [32].

In addition, confidence in the TMH recommendation on COVID-19 was independently associated with pediatricians’ recommendation of the COVID-19 vaccine for children. In other studies, trust in authorities’ healthcare guidelines was also found to be influencing parents’ decisions to vaccinate their children against COVID-19 [17,18]. A study conducted in the US has found that there was a decrease in the recommendation of both COVID-19 and routine vaccination by healthcare professionals throughout the pandemic, along with a decrease in trust in health authorities [35]. Continuous assessment of trust levels in health authorities and the impact on vaccination recommendations can inform public health strategies. Also, providing healthcare professionals with evidence-based information and involving them in the decision-making process regarding vaccination strategies is, therefore, essential, especially given that the majority of participants said that they recommend other vaccines that are not part of the national vaccination calendar to their patients.

### Study Strengths and Limitations

This study is the first of its kind in Tunisia. Several studies [36,37,38,39] were conducted before in the country to assess COVID-19 vaccine hesitancy, but none of them had a focus on the attitudes of pediatricians regarding COVID-19 vaccination in children or towards other vaccines that are not included in the NIS. Also, we conducted a national study with a random sample of pediatricians from both the private and the public sectors to ensure sample representativeness. Nevertheless, our study has some limitations that need to be mentioned. Indeed, this study was conducted in 2023, when the Omicron variant was the most frequently circulating strain. However, pediatricians’ attitudes may differ with changing infection rates and the emergence of new variants of concern (VOCs) with varying pathogenicity [22]. Also, we had a non-response rate of 41.8%, which can introduce a selection bias to the study, as those who did not accept to participate may be less likely to recommend the vaccine. Additionally, since the data collection tool was administered face-to-face, this approach could have led to desirability bias in the responses. Finally, we did not ask participants about their pediatric subspecialties, which may influence their willingness to recommend the COVID-19 vaccine for children.

## 5. Conclusions

While pediatricians are proactive in recommending certain non-NIS vaccines like the rotavirus and influenza vaccines for children with chronic diseases, there was notable hesitancy regarding the wide administration of the COVID-19 vaccine to children in July–October 2023. Health policymakers should include pediatricians in the decision-making process regarding vaccination for children strategies given their significant impact on parents’ decisions. Training programs tailored for pediatricians to emphasize the effectiveness and safety of newly developed vaccines for children should also be implemented. These initiatives will aim to fill knowledge gaps and enhance pediatricians’ confidence in recommending these vaccines, particularly in times of pandemics.

## Figures and Tables

**Table 1:** Sociodemographic characteristics of the surveyed pediatricians, Tunisia, 2023.

Variables	*n* (%)
Gender (N = 192)	
Male	82 (42.7)
Female	110 (57.3)
Age categories (years) (N = 190)	
≤40	47 (24.7)
41–60	95 (50.0)
≥60	48 (25.3)
Healthcare facility type (N = 192)	
Private sector	137 (71.3)
University hospital	37 (19.3)
Regional hospital	15 (7.8)
Primary healthcare facility	3 (1.6)
Professional experience (years) (N = 192)	
≤10	56 (29.2)
11–20	43 (22.4)
≥20	93 (48.4)
Region of practice (N = 192)	
North	128 (66.7)
Center	49 (25.5)
South	15 (7.8)
Having children under 16 years old (N = 192)	
Yes	135 (70.3)
No	57 (29.7)

**Table 2:** General statements related to the surveyed pediatricians’ attitudes regarding COVID-19 vaccines for children aged 16 and below, Tunisia, July–October 2023.

	n	Percentage (%)	[95% CI]
1. Vaccinating children against COVID-19 protects them from SARS-CoV-2 infection (N = 192)			
Agree	91	47.4	[40.3–54.5]
Neither agree nor disagree	14	7.3	[3.6–10.9]
Disagree	71	37.0	[30.1–43.8]
I don’t know	16	8.3	[4.4–12.2]
2. Vaccinating children against COVID-19 protects them from severe forms of the disease (N = 192)			
Agree	150	78.1	[72.3–84.0]
Neither agree nor disagree	8	4.2	[1.3–6.9]
Disagree	21	10.9	[6.5–15.3]
I don’t know	13	6.8	[3.2–10.3]
3. It is important to vaccinate children against COVID-19 to prevent its transmission to their families (N = 192)			
Agree	87	45.3	[38.3–52.3]
Neither agree nor disagree	14	7.3	[3.6–10.9]
Disagree	84	43.8	[36.7–50.8]
I don’t know	7	3.6	[0.9–6.3]
4. Vaccinating children against COVID-19 is important to reduce school absenteeism (N = 192)			
Agree	78	40.6	[33.7–47.6]
Neither agree nor disagree	16	8.3	[4.4–12.2]
Disagree	91	47.4	[40.3–54.5]
I don’t know	7	3.6	[0.9–6.3]
5. Vaccinating children against COVID-19 protects them from long COVID (N = 192)			
Agree	60	31.2	[24.7–37.8]
Neither agree nor disagree	13	6.8	[3.2–10.3]
Disagree	49	25.5	[19.3–31.7]
I don’t know	70	36.5	[29.6–43.3]
6. COVID-19 vaccine may cause serious side effects in children (N = 192)			
Agree	88	45.8	[38.8–52.9]
Neither agree nor disagree	12	6.3	[2.8–9.7]
Disagree	32	16.7	[11.4–21.9]
I don’t know	60	31.3	[24.7–37.8]
7. Natural immunity following COVID-19 infection lasts longer than acquired immunity following vaccination (N = 191)			
Agree	84	44.0	[36.9–51.0]
Neither agree nor disagree	5	2.6	[0.3–4.9]
Disagree	46	24.1	[18.0–30.1]
I don’t know	56	29.3	[22.9–35.8]

CI: confidence interval.

**Table 3:** Factors significantly associated with pediatricians’ recommendation of the COVID-19 vaccine to children aged 16 and below: results of the univariate analysis, Tunisia, July–October 2023.

Variables	N	Recommend (%)	Do not Recommend (%)	Crude OR [95% CI]	*p*
Age categories (years)					*p* = 0.008
≤40	47	59.6	40.4	2.5 [1.1–5.6]	
41–60	95	32.6	67.4	0.8 [0.4–1.7]	
≥60	48	37.5	62.5	1	
Sector of practice					
Public	55	56.4	43.6	2.5 [1.3–4.7]	*p* = 0.005
Private	137	34.3	65.7	1	
Having children under 16 years old					*p* = 0.012
No	57	54.4	45.6	2.2 [1.2–4.2]	
Yes	135	34.8	65.2	1	
Vaccinating children against COVID-19 protects them from SARS-CoV-2 infection					
Agree	91	48.4	51.6	1.8 [1.1–3.3]	*p* = 0.039
Other *	101	33.7	66.3	1	
Vaccinating children against COVID-19 protects them from severe forms of the disease					
Agree	150	49.3	50.7	9.2 [3.1–27.2]	*p* < 10^–3^
Other *	42	9.5	90.5	1	
It is important to vaccinate children against COVID-19 to prevent its transmission to their families					
Agree	87	57.5	42.5	3.7 [2.0–6.8]	*p* < 10^–3^
Other *	105	26.7	73.3	1	
Vaccinating children against COVID-19 is important to reduce school absenteeism					
Agree	78	60.3	39.7	4.1 [2.2–7.5]	
Other *	114	27.2	72.8	1	*p* < 10^−3^
Vaccinating children against COVID-19 protects them from long COVID					
Agree	60	51.7	48.3	1.9 [1.1–3.6]	*p* = 0.036
Other *	132	35.6	64.4	1	
COVID-19 vaccine may cause serious side effects in children					
Agree	88	29.5	70.5	1	*p* = 0.004
Other *	104	50.0	50.0	2.4 [1.3–4.3]	
Pediatricians’ confidence level in the TMH recommendations on COVID-19 vaccination					
Medium/High confidence	140	50.7	49.3	9.1 [3.4–24.2]	*p* < 10^−3^
No/Limited confidence	49	10.2	89.9	1	
Pediatricians’ confidence level in WHO recommendations on COVID-19 vaccination					
Medium/High confidence	131	52.7	47.3	8.3 [3.5–19.5]	*p* < 10^−3^
No/Limited confidence	59	11.9	88.1	1	

WHO: World Health Organization. TMH: Tunisian Ministry of Health. CI: confidence interval. OR: odds ratio. *: Other includes “Disagree”, “Neutral”, and “I don’t know”.

**Table 4:** Factors associated with pediatricians’ recommendation of the COVID-19 vaccine to children aged 16 and below: results of the final model of the multivariable analysis, Tunisia, July–October 2023.

Associated Factors	Adjusted OR	[95% CI]
Pediatricians’ confidence level in the TMH recommendations on COVID-19 vaccination		
Medium/High confidence	6.1	[2.2–16.9]
No/Limited confidence	1	
Vaccinating children against COVID-19 is important to reduce school absenteeism		
Agree	2.3	[1.1–5.1]
Other *	1	

TMH: Tunisian Ministry of Health. *: Other includes “Disagree”, “Neutral”, and “I don’t know”.

## Data Availability

The datasets used and/or analyzed during the current study are available from the corresponding author on reasonable request.

## References

[1] Global Immunization Efforts Have Saved at Least 154 Million Lives over the Past 50 Years. https://www.who.int/news/item/24-04-2024-global-immunization-efforts-have-saved-at-least-154-million-lives-over-the-past-50-years.

[2] Global Vaccination Schedules. https://vaccineknowledge.ox.ac.uk/vaccination-schedules-other-countries.

[3] (2024). Vaccination Schedules in the EU/EEA. https://vaccination-info.europa.eu/en/about-vaccines/when-vaccinate/vaccination-schedules-eueea.

[4] Ministère de la Santé Publique—Ministère de la Santé Publique. https://sante.gouv.fr/.

[5] Immunization Data WHO Immunization Data Portal—Eastern Mediterranean Region. https://immunizationdata.who.int/dashboard/regions/eastern-mediterranean-region.

[6] Interim Recommendations for Use of the Moderna mRNA-1273 Vaccine Against COVID-19. https://iris.who.int/bitstream/handle/10665/361718/WHO-2019-nCoV-vaccines-SAGE-recommendation-mRNA-1273-2022.2-eng.pdf.

[7] Children and COVID-19 Vaccines—Parents’ Questions Answered|UNICEF South Asia. https://www.unicef.org/rosa/stories/children-and-covid-19-vaccines.

[8] Tunisie: Campagne de Vaccination Contre le Coronavirus au Profit des Enfants Souffrant de Maladies Chroniques. https://www.aa.com.tr/fr/afrique/tunisie-campagne-de-vaccination-contre-le-coronavirus-au-profit-des-enfants-souffrant-de-maladies-chroniques/2676125.

[9] Gnet News (2021). Tunisie-Coronavirus: Démarrage de la Vaccination des Enfants de 12 à 14 Ans. https://news.gnet.tn/lancement-de-la-vaccination-des-enfants-de-12-a-14-ans/.

[10] Portail National de Vaccination. https://vaccination-info-service.fr/.

[11] Yalçin S.S., Bakacak A.G., Topaç O. (2020). Unvaccinated children as community parasites in National Qualitative Study from Turkey. BMC Public Health.

[12] Choi S.-H., Jo Y.H., Jo K.J., Park S.E. (2021). Pediatric and Parents’ Attitudes Towards COVID-19 Vaccines and Intention to Vaccinate for Children. J. Korean Med. Sci..

[13] Hetherington E., Edwards S.A., MacDonald S.E., Racine N., Madigan S., McDonald S., Tough S. (2021). SARS-CoV-2 vaccination intentions among mothers of children aged 9 to 12 years: A survey of the All Our Families cohort. CMAJ Open.

[14] Leib S., Liberatos P., Edwards K. (2011). Pediatricians’ Experience with and Response to Parental Vaccine Safety Concerns and Vaccine Refusals: A Survey of Connecticut Pediatricians. Public Health Rep..

[15] Karafillakis E., Dinca I., Apfel F., Cecconi S., Wűrz A., Takacs J., Suk J., Celentano L.P., Kramarz P., Larson H.J. (2016). Vaccine hesitancy among healthcare workers in Europe: A qualitative study. Vaccine.

[16] Al-Jayyousi G.F., Sherbash M.A.M., Ali L.A.M., El-Heneidy A., Alhussaini N.W.Z., Elhassan M.E.A., Nazzal M.A. (2021). Factors Influencing Public Attitudes towards COVID-19 Vaccination: A Scoping Review Informed by the Socio-Ecological Model. Vaccines.

[17] Steletou E., Giannouchos T., Karatza A., Sinopidis X., Vervenioti A., Souliotis K., Dimitriou G., Gkentzi D. (2022). Parental and Pediatricians’ Attitudes towards COVID-19 Vaccination for Children: Results from Nationwide Samples in Greece. Children.

[18] Bourguiba A., AbuHijleh S., Nached Y., Waleed D., Farghaly S., AlOlama F. (2023). Assessing Parents’ Knowledge, Attitudes, and Practices Toward Vaccinating Children (Five to 15 Years Old) Against COVID-19 in the United Arab Emirates. Cureus.

[19] Pan F., Zhao H., Nicholas S., Maitland E., Liu R., Hou Q. (2021). Parents’ Decisions to Vaccinate Children against COVID-19: A Scoping Review. Vaccines.

[20] Al-Qerem W., Al Bawab A.Q., Hammad A., Jaber T., Khdair S.I., Kalloush H., Ling J., Mosleh R. (2022). Parents’ attitudes, knowledge and practice towards vaccinating their children against COVID-19: A cross-sectional study. Hum. Vaccines Immunother..

[21] Kocamaz E.B., Kocamaz H. (2022). Awareness of COVID-19 and attitudes toward vaccination in parents of children between 0 and 18 years: A cross-sectional study. J. Pediatr. Nurs..

[22] Ali Y., Piche-Renaud P.-P., Karimi-Shahrbabak E., Farrar D.S., Fadaleh S.A., Burey S., Morris S.K. (2023). Pediatricians’ perceptions, practices, and barriers regarding COVID-19 vaccine for children: A cross-sectional survey in Ontario, Canada. Vaccine.

[23] Elitok G.K., Koc A., Apaydin S., Dincer B.T., Bulbul A. (2024). Knowledge, Attitudes and Practices of Pediatricians About COVID-19 Vaccination to Children. Med. Bull. Sisli Etfal Hosp..

[24] Alfaqih M.A., Ababneh E.Y., Almansi G.A., Marashdeh S.A., Khazandar A.A., Said A.M., Mustafa A.G. (2023). The attitude and knowledge of pediatricians and family physicians toward COVID-19 vaccination in children: A cross-sectional study. Hum. Vaccines Immunother..

[25] Pan W.K.M. (2024). The application of behavioral change theories in addressing vaccine hesitancy: A Literature Review. Public Health Nurs..

[26] Çataklı T., Duyan-Çamurdan A., Aksakal-Baran F.N., Güven A.E., Beyazova U. (2018). Attitudes of physicians concerning vaccines not included in the national immunization schedule. Turk. J. Pediatr..

[27] Soltani M.S., Salah A.B., Bouanene I., Trabelsi A., Sfar M.T., Harbi A., Gueddiche M.N., Farhat E.B. (2015). Epidemiology and medical cost of hospitalization due to rotavirus gastroenteritis among children under 5 years of age in the central-east of Tunisia. EMHJ-East. Mediterr. Health J..

[28] Immunization Data WHO Immunization Data Portal—Detail Page. https://immunizationdata.who.int/global/wiise-detail-page.

[29] Dbaibo G., Tatochenko V., Wutzler P. (2016). Issues in pediatric vaccine-preventable diseases in low- to middle-income countries. Hum. Vaccines Immunother..

[30] Meeting of the Strategic Advisory Group of Experts on Immunization, April 2012: Conclusions and Recommendations. https://www.who.int/publications/i/item/WER8721.

[31] CDC Influenza (Flu). Flu and Children. https://www.cdc.gov/flu/highrisk/children.html.

[32] WHO SAGE Roadmap for Prioritizing Uses of COVID-19 Vaccines: An Approach to Optimize the Global Impact of COVID-19 Vaccines, Based on Public Health Goals, Global and National Equity, and Vaccine Access and Coverage Scenarios. https://www.who.int/publications/i/item/WHO-2019-nCoV-Vaccines-SAGE-Prioritization-2023.1.

[33] Pierce C.A., Herold K.C., Herold B.C., Chou J., Randolph A., Kane B., McFarland S., Gurdasani D., Pagel C., Hotez P. (2022). COVID-19 and children. Science.

[34] Updated WHO SAGE Roadmap for Prioritizing Uses of COVID-19 Vaccines. https://www.who.int/news/item/21-01-2022-updated-who-sage-roadmap-for-prioritizing-uses-of-covid-19-vaccines.

[35] Dudley M.Z., Schuh H.B., Forr A., Shaw J., Salmon D.A. (2024). Changes in vaccine attitudes and recommendations among US Healthcare Personnel during the COVID-19 pandemic. npj Vaccines.

[36] Dhouib W., Fredj M.B., Bennasrallah C., Gara A., Maatouk A., Zemni I., Abroug H., Kacem M., Bouanene I., Sriha A.B. (2023). COVID-19 Vaccine Hesitancy Among a Sample of Students in Tunisia. Popul. Med..

[37] Zammit N., Gueder A.E., Brahem A., Ayouni I., Ghammam R., Fredj S.B., Sridi C., Chouchene A., Kalboussi H., Maalel O.E. (2022). Studying SARS-CoV-2 vaccine hesitancy among health professionals in Tunisia. BMC Health Serv. Res..

[38] Maatouk A., Ammar A., Ezzi O., Chelly S., Bannour W., Helali R., Njah M., Mahjoub M. (2022). Hesitation of the Tunisian population to COVID-19 vaccination, and its associated factors. Tunis. Médicale.

[39] Khiari H., Cherif I., M’ghirbi F., Mezlini A., Hsairi M. (2021). COVID-19 Vaccination Acceptance and Its Associated Factors among Cancer Patients in Tunisia. Asian Pac. J. Cancer Prev. APJCP.

